# Experimental Study on the Size Effect of Compression-Shear Fracture Characteristics of Rock-like Materials Containing Open Cracks

**DOI:** 10.3390/ma17235941

**Published:** 2024-12-04

**Authors:** Zixuan Li, Shiyuan Huang, Chuan Lv, Cheng Liao, Xudong Li, Hongbo Du

**Affiliations:** 1 Engineering Research Center of Diagnosis Technology and Instruments of Hydro-Construction, Chongqing Jiaotong University, Chongqing 400074, China; 13036362198@163.com (Z.L.); cqjt1293@163.com (C.L.); cqjtdxlxd@163.com (X.L.); duhongbo@cqjtu.edu.cn (H.D.); 2 School of Materials Science and Engineering, Chongqing Jiaotong University, Chongqing 400074, China; 3 Jiangxi Academy of Water Science and Engineering, Nanchang 330029, China; chengliaomiao@163.com

**Keywords:** quasi-brittle materials, compression-shear, open crack, crack inclination, crack initiation angle

## Abstract

Understanding fracture mechanics in rock-like materials under compression-shear condition is critical for predicting failure mechanisms in various engineering applications, such as mining and civil infrastructure. This study conducted uniaxial compression tests on cubic gypsum specimens of varying sizes (side lengths of 75 mm, 100 mm, 125 mm, and 150 mm) and crack inclination angles (ranging from 0° to 90°) to assess the size effect on fracture behavior. The effects of specimen size and crack inclination on fracture characteristics, including strength, failure mode, and crack initiation angle, were analyzed based on the maximum tangential stress (MTS) criterion and the generalized maximum tangential stress (GMTS) criterion, with relative critical size (α) and relative openness (*η*). Results indicate that the crack initiation angle increases with crack inclination, while compressive strength decreases significantly with increasing specimen size. For example, at a 30° crack inclination, the peak compressive strength of 75 mm specimens was 2.53 MPa, whereas that of 150 mm specimens decreased to 1.05 MPa. Crack type and failure mode were found to be primarily influenced by crack inclination rather than specimen size. The experimental crack initiation angle aligned with the theoretical crack initiation angle at inclinations below 50° but diverged at higher inclinations. A linear relationship was established between *r*_c_ and specimen size (*L*) under compression-shear stress, expressed as $r_{c}=-0.01772L+3.54648$; larger specimens exhibited increased tangential stress at the crack tip, leading to earlier macroscopic crack formation, while *r*_c_ decreased as specimen size increased. These results underscore the significant influence of size on fracture behavior in quasi-brittle materials under compression-shear stress, providing essential insights for predicting material failure in rock-like structures.

## 1. Introduction

Since the introduction of fracture mechanics theory into geomechanics, the crack initiation strength and crack propagation in rock materials has received a lot of attention [1,2,3,4,5,6]. From the perspective of load, the external load on the crack can be converted into normal and tangential forces, thus turning the question into the fracture problem under the action of pure tension, pure shear, tension-shear, and compression-shear [7,8,9,10,11]. Since the mechanical environments of engineering rock masses are mostly compressive, studying the compression-shear fracture problem is of practical significance.

When compression-shear cracks in an engineering rock mass are closed, the stress field at the crack tip is governed by the mode II stress intensity factor *K*_II_ [12,13,14,15]. Under the maximum tangential stress (MTS) criterion in linear elastic fracture mechanics, the tension crack initiation angle is consistently 70.53°. Extensive tests on rock materials have shown that regardless of whether cracks are open or closed, the tension crack initiation angle varies with changes in the pre-set crack inclination angle [16,17,18,19]. This significant difference from the theoretical value cannot be explained by the traditional theory. Researchers have suggested that the non-singular constant term (T-stress) ignored in the Williams expansion in traditional fracture mechanics impacts the calculation results [20,21]. Studies have shown that simultaneously considering the singular stress term and the non-singular constant term at the crack tip can greatly improve calculation results [22,23,24]. Initially introduced to analyze fractures in brittle materials under tension-shear, T-stress has been recently applied to the compression-shear fracture of rock materials, leading to the development of criteria for closed cracks that incorporate T-stress [25,26,27,28]. When T-stress is considered, the initiation angle of a closed crack increases consistently with the prefabricated crack inclination angle. Moreover, the crack initiation load and angle are strongly influenced by fracture performance and the fracture process zone size in brittle specimens of equal crack length [19,29,30,31]. The brittleness of the material substantially influences its behavior, especially in regions with structural defects. As the fracture process zone of rock-like materials is larger than that of conventional brittle materials, like ceramics [32,33,34], T-stress exerts a more significant influence on the fracture behavior of rock-like materials.

Open cracks are common, be they engineering rock mass cracks or specimen prefabricated cracks. Previous researchers hold different views on treating the mode I stress intensity factor *K*_I_ for compression-shear cracks [35] (*K*_I_ < 0 and *K*_I_ = 0). For closed cracks, there is no doubt that *K*_I_ = 0, while *K*_I_ < 0 essentially stipulates that compression stress has an inhibitory effect on crack opening. Regarding the initiation of open cracks, Xu et al. [36] pointed out that under the action of compressive-shear stress, the prefabricated crack tip expands first, and under uniaxial compression, the tensile crack is mostly generated and expands along the direction of the maximum compressive stress. However, under biaxial compression, random expanding shear cracks are generated. Lin et al. [37] pointed out that due to the different crack prefabrication methods, some prefabricated closed cracks under laboratory conditions are not really closed cracks but have a certain degree of relative openness, leading to large discrepancies when analyzing the initiation of open cracks using the theoretical model of closed cracks. For the open cracks, the calculation result is fixed because the traditional theory abstracts it as mathematical cracks without thickness, while the different geometric characteristics of different prefabricated cracks (e.g., crack length, crack thickness, and crack tip curvature radius) are important reasons for the discrete tension crack initiation angles.

In addition, assessing the mechanical properties of real engineering rock masses is highly complex, costly, and time intensive. Consequently, researchers often conduct small-scale experiments in the laboratory and then extend the experimental results to actual engineering. However, the fracture process zone is typically not sufficiently small in relation to the specimen, as laboratory specimens are generally small, which often leads to significant size effects in experimental results [38,39,40,41]. Extensive research has been conducted on the size effects of fractures in various rock materials [42,43]. For instance, Zdeněk P. Bažant [44] reviews the size effect on the nominal strength of structures, exploring the transition from statistical models to energy-based approaches and their application to fracture mechanics, plasticity, and quasi-brittle materials. Elena Ferretti [45] introduces a discrete nonlocal formulation for fracture mechanics, enhancing the modeling of stress distribution and crack propagation. Saouma et al. [46] introduced a new method for size effect based on the local stress intensity factor, revealing that size effects occur not only in quasi-brittle materials but also in elastoplastic materials. Karamloo [47] investigated the size effect of various crack lengths under specific geometries. Hu et al. [48] demonstrated that the mechanism of the size effect in concrete materials originates from the microcrack region surrounding the macrocrack tip, known as the fracture process zone (FPZ). When the FPZ interacts with the specimen boundary, size effects arise. Wang et al. [49] examined the size effect using a meso notched beam and found that the relative influence of the boundary effect on the FPZ decreased as sample size increased. They also established a functional relationship between nominal strength, FPZ length, and notched beam height. However, most existing research primarily addresses the size effect in mode I and I-II mixed-mode fracture characteristics, with relatively few studies on the size effect of compression-shear fracture characteristics in rock-like materials. For instance, Hu et al. [50] developed a numerical model of rock samples with large defects under compression using PFC2D software demonstrating that specimen size significantly influences the crack characteristics, mechanical properties, and energy properties of the rock. Jin et al. [51]. used 3D modeling to simulate the mechanical response of cubic concrete specimens of varying sizes under dynamic biaxial compression with different strain rates and stress ratios. They derived a formula for the size effect that quantitatively accounts for the influence of strain rate and lateral stress. Wang et al. [18]. conducted uniaxial compression tests on gypsum specimens of various sizes, revealing that larger specimens have smaller fracture process zones (FPZs) at the crack tip.

In view of this, this study prepares cubic gypsum specimens of 4 sizes and 10 crack inclination angles, observes the open crack propagation process through unconfined uniaxial compression tests, and analyzes the size effects on specimen failure load and crack initiation angle. Additionally, the relative critical size *α* is introduced to characterize the material properties while relative openness *η* describes the geometric attributes of open cracks. The compression-shear fracture criterion considering T-stress is developed for materials with open cracks. The criterion’s applicability is verified through comparison with the experimental results of this study. Finally, the criterion is used to calculate the critical crack tip size (fracture process zone size) for specimens of different sizes. Results indicate that, under the compression-shear stress, the fracture process zone at the crack tip of rock-like materials decreases with increasing specimen size, in contrast to the pattern under mode I loading and I-II mixed-mode loading.

## 2. Test Materials and Methods

### 2.1. Specimen Preparation

**Figure 1:**
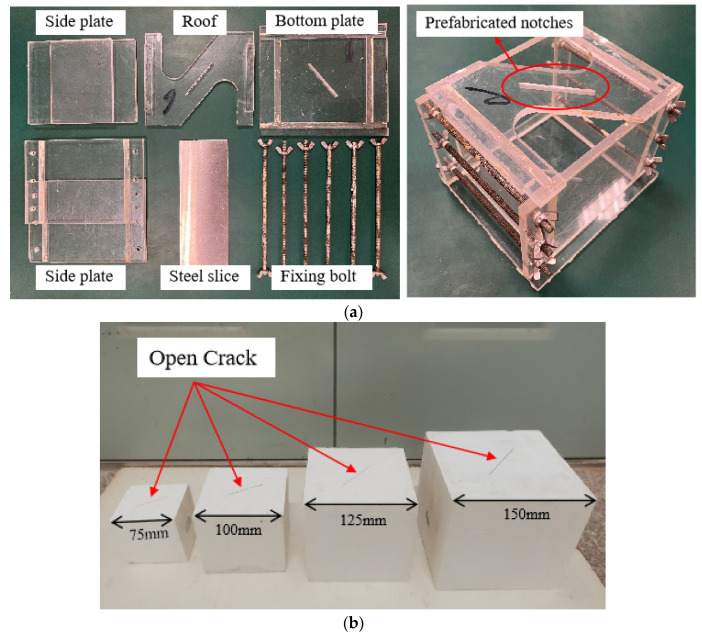
Gypsum specimen preparation device and prepared specimens: (**a**) the assembled device for preparing gypsum specimens with open cracks; (**b**) gypsum specimens of different sizes and open cracks.

Cubic gypsum specimens were prepared for the tests. This material has similar structural and failure characteristics to rocks and is characterized by stable mechanical properties, easy fabrication, and low cost, so it is commonly used in the study of rock-like materials [52,53]. As shown in Figure 1a, the preparation device for gypsum specimens with open cracks consists of a bottom plate, side plates, a top plate (roof), fixing bolts, and a steel sheet. Among them, the fixing bolts and the steel sheet are metal, and the other parts are tempered glass. The steel sheet is 1 mm thick. The test materials were desulfurized gypsum powder and laboratory tap water. Briefly, the gypsum powder and water were evenly mixed according to a mass ratio of 1:1 and poured into the assembled cubic gypsum specimen preparation device. After curing at 20 °C ± 2 °C for 1 h to 2 h, the steel sheet wiped with engine oil was inserted into the specimen through the prefabricated slit on the top plate. All specimens were demolded after 24 h, and the steel sheet was removed from the specimen. The crack penetration was checked to ensure successful open crack prefabrication in the center. The prepared specimens were transferred into a curing box to cure for 28 days at 20 °C ± 2 °C according to the standard curing method. After curing, the specimen surface was ground to reduce the impact of friction while facilitating observation of the whole crack initiation and expansion process in the subsequent uniaxial compression tests. Figure 1b shows four open crack gypsum specimens of different sizes after polishing.

### 2.2. Test Protocol

Four test groups were designed to analyze the mechanical properties and failure behaviors of open crack specimens. Specifically, the ratio of the prefabricated crack length (2*a*) to the specimen side length (*L*) was 2*a*/*L* = 0.5, the prefabricated crack thickness (2*b*) was 1 mm, and the specimen side length (*L*) and the prefabricated crack inclination (*β*) were variables. The detailed experimental setup is presented in Table 1. The specimen side lengths *L* were from 75 mm to 150 mm, with prefabricated crack inclination (*β*) ranges from 0° to 90°, with an interval of 10 (i.e., 0°, 10°, 20°, 30°, 40°, 50°, 60°, 70°, 80°, and 90°). Three parallel experiments were conducted for each test group to ensure the accuracy of the test results, with a total of 120 specimens.

### 2.3. Loading Method

**Figure 2:**
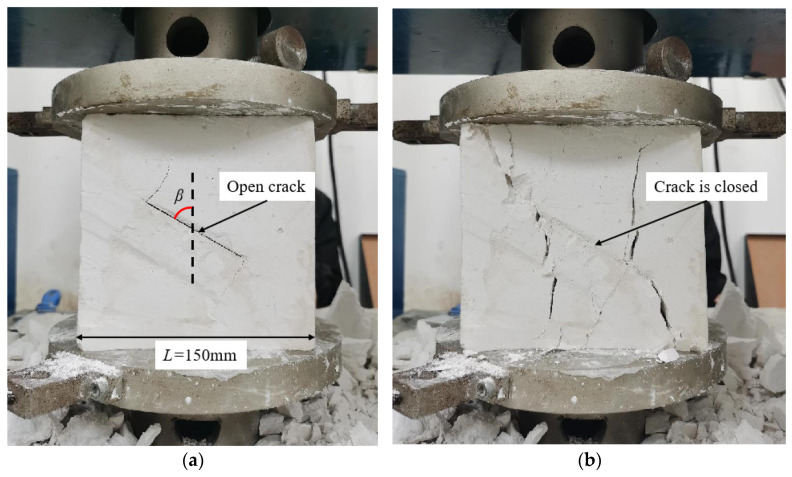
Specimen loading and failure patterns: (**a**) the condition of the specimen during the initial loading stage; (**b**) the condition of the specimen at final failure.

Uniaxial compression tests were conducted using an electro-hydraulic servo control device with a maximum load capacity of 500 kN. Vaseline was applied to the plates to minimize friction before loading. The system applied displacement-controlled loading at a rate of 0.8 mm/min until specimen failure occurred, with vertical displacement and load data automatically recorded by the supporting software, while a high-speed camera captured the full crack initiation and propagation process. Figure 2 shows a gypsum specimen with an open crack, measuring *L* = 150 mm and a prefabricated crack inclination of *β* = 60° under uniaxial compression testing. In Figure 2a, significant crack propagation was observed at both ends of the prefabricated crack during the early loading stages, with smooth, parallel propagation paths. With continued loading, the open crack gradually closed. Figure 2b shows the specimen at final failure, where the crack had propagated through the entire specimen, and the prefabricated crack appeared closed.

## 3. Compression-Shear Fracture Theory of Rock-like Materials

### 3.1. Stress Field at the Open Crack Tip

**Figure 3:**
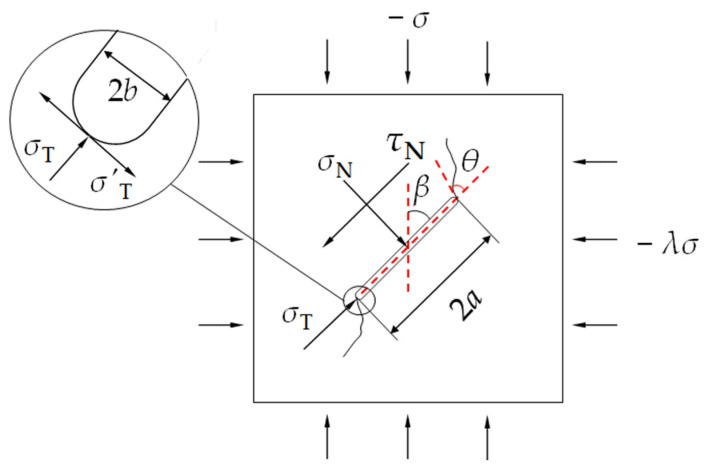
Stress state of the open crack on the upper surface.

To better analyze the stress state of the crack on the lateral surface of the specimen as shown in Figure 2a, within the framework of traditional fracture mechanics theory and based on linear elasticity, a model of a central open inclined crack under biaxial compression conditions was developed, as illustrated in Figure 3. The crack length is 2*a*, thickness is 2*b*, and the prefabricated crack inclination (*β*) is defined as the angle between the crack and vertical direction. The crack was subjected to far-field stresses, with the principal stress (−$\sigma_{T}$) and lateral stress (−*λσ*) acting on the crack where tensile stress is defined as positive and compressive stress as negative. $\sigma_{N}$ is the normal stress perpendicular to the prefabricated crack, $\tau_{N}$ is the shear stress parallel to the prefabricated crack, and $\sigma_{T}$ is the tangential stress when the crack is open. The parameter *λ* is the lateral compression coefficient [54,55]. At the crack tip, the strain in the thickness direction is sufficiently constrained, allowing the crack tip region to be approximated as a plane strain state. This model primarily analyzes the stress field on the surface of cubic cracks and does not consider internal cracks in specimens with finite thickness. While this simplification facilitates the analysis and provides a useful foundation for understanding the crack behavior in cubic specimens, it has certain limitations when analyzing the failure behavior within the specimen. According to Muskhelishvili’s [56] research, since an open crack has a certain thickness, it is necessary to consider the lateral compressive stress $\sigma_{T}$ parallel to the crack. This lateral stress generates a tensile stress ${\sigma^{\prime }}_{T}$ at the crack tip, equal in magnitude to $\sigma_{T}$. This tensile stress has a significant impact on crack propagation, especially under tensile action at the crack tip, where it notably influences the crack propagation mode.

To more accurately describe the geometric characteristics of open cracks, the crack tip can be simplified as a semi-circular shape with a radius of *b*, as shown in the enlarged detail of the crack tip in Figure 3. This approximation better matches the geometric characteristics of cracks in real engineering applications, especially in brittle materials such as rock and gypsum, where open cracks typically exhibit this type of tip structure. The stress state of the crack on the upper surface is described as follows:

(1) $$\begin{array}{l} \sigma_{T}=-\sigma (\cos^{2}\beta +\lambda \sin^{2}\beta )\\ \sigma_{N}=-\sigma (\sin^{2}\beta +\lambda cos^{2}\beta )\\ \tau_{N}=-\sigma (1-\lambda )\sin\beta \cos\beta \end{array}\},$$

**Figure 4:**
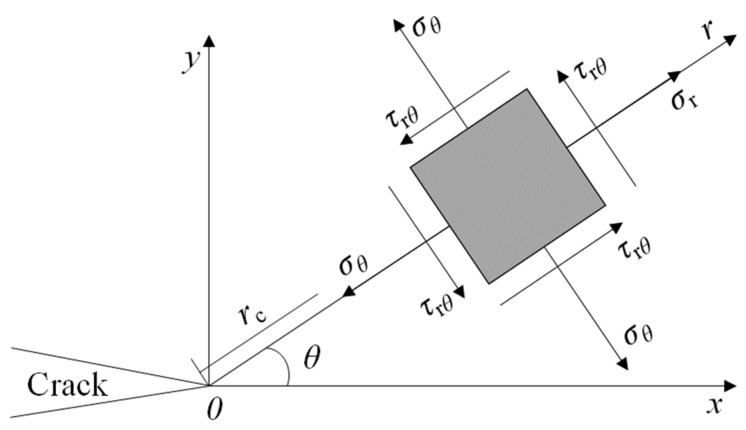
Stress fields at crack tip.

According to the traditional fracture theory, the stress field at the tip of the classical I-II mixed-mode crack is shown in Figure 4. The stress components can be divided into radial stress $\sigma_{r}$, circumferential stress $\sigma_{\theta }$, and shear stress $\tau_{r\theta }$.

The stress components in the figure are represented by polar coordinates as

(2) $$\begin{array}{l} \sigma_{\theta }=\frac{1}{2\sqrt{2\pi r}}\cos\frac{\theta }{2}\left[K_{I}\left(1+\cos\theta \right)-3K_{\mathrm{II}}\sin\theta \right]\\ \sigma_{r}=\frac{1}{2\sqrt{2\pi r}}\left[K_{\mathrm{II}}\sin\frac{\theta }{2}\left(3\cos\theta -1\right)+K_{I}\cos\frac{\theta }{2}\left(3-\cos\theta \right)\right]\\ \tau_{r\theta }=\frac{1}{2\sqrt{2\pi r}}\cos\frac{\theta }{2}\left[K_{\mathrm{II}}\left(3\cos\theta -1\right)+KI\;\sin\theta \right] \end{array}\},$$

where *r* is the distance from the crack tip to the element, and *θ* is the angle at which the element deviates from the crack. *K*_I_ and *K*_II_ are stress intensity factors (SIFs) for Mode I and Mode II cracks, reflecting the intensity of the stress field around the crack tip.

For open cracks, both the Mode I stress intensity factor *K*_I_*^T^* and *K*_I_*^N^*, generated by the tensile stress ${\sigma^{\prime }}_{T}$ and the normal stress $\sigma_{N}$ must be considered as follows:

(3) $$\begin{array}{l} K_{I}^{T}=-0.5\sigma_{T}\sqrt{b/a}\sqrt{\pi b}\\ K_{I}^{N}=\sigma_{N}\sqrt{\pi b} \end{array}\},$$

According to Equation (3), crack thickness and length impact *K*_I_. For comprehensiveness, the thickness and length in Equation (3) can be normalized for quantitative descriptions. $\eta =\sqrt{b/a}$ is defined as the relative openness describing the *K*_I_ under different crack length and thickness combinations, and as relative openness *η* approaches 0, it corresponds to the ideal conditions for mathematical cracks.

In summary, *K*_I_ on the open crack is affected by the combination of ${\sigma^{\prime }}_{T}$ and $\sigma_{N}$, which can be expressed as follows:

(4) $$K_{I}=K_{I}^{T}+K_{I}^{N}=-\sigma \sqrt{\pi b}\left[\left(sin^{2}\beta +\lambda cos^{2}\beta \right)-0.5\left(cos^{2}\beta +\lambda sin^{2}\beta \right)\eta \right],$$

In contrast, *K*_II_ on the open crack is affected by the shear stress $\tau_{N}$, which can be expressed as the following:

(5) $$K_{\mathrm{II}}=\tau_{N}\sqrt{\pi b}=-\sigma (1-\lambda )\sin\beta \cos\beta \sqrt{\pi b}.$$

### 3.2. Crack Extension Criterion Considering T-Stress

Using traditional fracture mechanics theory, Williams et al. described the stress field at the crack tip through an orthogonal series expansion, with each term reflecting different aspects of the stress field [57]:

(6) $$\sigma_{ij}=A_{1}r^{-0.5}f_{ij}^{1}(\theta )+A_{2}r^{0}f_{ij}^{2}(\theta )+A_{3}r^{0.5}f_{ij}^{3}(\theta )+\ldots ,$$

where the first term is the singular stress term, which dominates at the crack tip, with the coefficient *A*_1_ known as the stress intensity factor; the second term is a non-singular term, commonly referred to as *T* stress, which is a constant term independent of *r*; and the third and subsequent items are higher-order items which can be ignored as *r* approaches 0.

In laboratory tests, shear cracks are often observed after tensile cracks or not observed at all. Therefore, numerous compression-shear fracture criteria are mainly based on the study of tensile cracking [52,58]. Among these, the maximum tangential stress criterion (MTS criterion), first proposed by Erdogan and Sih [59],is used to predict the tension crack initiation angle and propagation direction. It has become the most widely adopted criterion due to its clear meaning and minimal reliance on influencing factors. The MTS criterion is based on the two assumptions: cracks do not begin to propagate until reaching the maximum tangential stress and cracks begin to propagate in the maximum tangential stress direction, and the MTS criterion can be described based on the following mathematical equation:

(7) $$\begin{array}{l} {\frac{\partial \sigma_{\theta }}{\partial \theta }|}_{r=r_{c}}=0\Rightarrow \theta =\theta_{0}\\ {\frac{\partial^{2}\sigma_{\theta }}{\partial \theta^{2}}|}_{r=r_{c}}<0 \end{array},$$

Previous studies only retained the singular items and ignored the non-singular constant items. However, the critical crack tip size *r*_c_ or the fracture process zone (FPZ) size, in rocks or rock-like materials is larger than, in general, brittle materials, leading to a large difference between the tension crack initiation angle and the theoretical value. Thus, the influence of both the constant and non-singular terms must be considered [32,33]. Based on the MTS criterion, Smith et al. [27] proposed the generalized maximum tangential stress criterion (GMTS) that considered the effect of T-stress. Previous study indicated that, under compression-shear stress, the T-stress (*T_x_*) parallel to the crack surface should be considered for open cracks [60]. The stress field at the open crack tip, accounting for T-stress, can be expressed as

(8) $$\begin{array}{l} \sigma_{\theta }=\frac{1}{2\sqrt{2\pi r}}\cos\frac{\theta }{2}\left[K_{I}\left(1+\cos\theta \right)-3K_{\mathrm{II}}\sin\theta \right]+T_{x}\sin^{2}\theta \\ \sigma_{r}=\frac{1}{2\sqrt{2\pi r}}\left[K_{I}\cos\frac{\theta }{2}\left(3-\cos\theta \right)+K_{\mathrm{II}}\sin\frac{\theta }{2}\left(3\cos\theta -1\right)\right]+T_{x}\cos^{2}\theta \\ \tau_{r\theta }=\frac{1}{2\sqrt{2\pi r}}\cos\frac{\theta }{2}\left[K_{I}\sin\theta +K_{\mathrm{II}}\left(3\cos\theta -1\right)\right]-\frac{1}{2}T_{x}\sin2\theta \end{array}\},$$

where

(9) $$T_{x}=-\sigma (\cos^{2}\beta +\lambda \sin^{2}\beta ),$$

Substituting the first equation from Equation (8) into Equation (7) yields the maximum tangential stress at the open crack tip, accounting for *T*-stress, as follows:

(10) $$\begin{array}{l} {\left(\sigma_{\theta }\right)}_{\max}=\frac{1}{2\sqrt{2\pi r_{c}}}\cos\frac{\theta_{0}}{2}\{-\sigma \sqrt{\pi a}\left[\begin{array}{l} \left(\sin^{2}\beta +\lambda \cos^{2}\beta \right)\\ -0.5\left(\cos^{2}\beta +\lambda \sin^{2}\beta \right)\eta \end{array}\right]\cdot \left(1+\cos\theta_{0}\right)\\ +3\sigma \sqrt{\pi a}\left(1-\lambda \right)\sin\beta \cos\beta \sin\theta_{0}\}-\sigma \left(\cos^{2}\beta +\lambda \sin^{2}\beta \right)\sin^{2}\theta_{0} \end{array},$$

It can be seen that after accounting for T stress, both *r_c_* and crack half-length (*a*) will influence the crack initiation angle and the maximum circumferential stress. For simplicity in analysis, the relationship between *r_c_* and crack length is normalized by introducing a relative critical size $\alpha =\sqrt{2r_{c}/a}$. Equation (10) can be simplified as follows:

(11) $$\begin{array}{l} {\left(\sigma_{\theta }\right)}_{\max}=\frac{1}{2\alpha }\cos\frac{\theta_{0}}{2}\{-\sigma \left[\left(\sin^{2}\beta +\lambda \cos^{2}\beta \right)-0.5\left(\cos^{2}\beta +\lambda \sin^{2}\beta \right)\eta \right]\cdot \\ (1+\cos\theta_{0})+3\sigma (1-\lambda ){\sin\beta \cos\beta \sin\theta }_{0}\}-\sigma \left(\cos^{2}\beta +\lambda \sin^{2}\beta \right)\sin^{2}\theta_{0} \end{array}$$

This equation describes the geometric characteristics of the crack through the relative openness *η* and considers the influence of material properties through the relative critical crack tip size *α*.

## 4. Test Results Analysis

### 4.1. Compressive Strength of Open Crack Specimens

**Figure 5:**
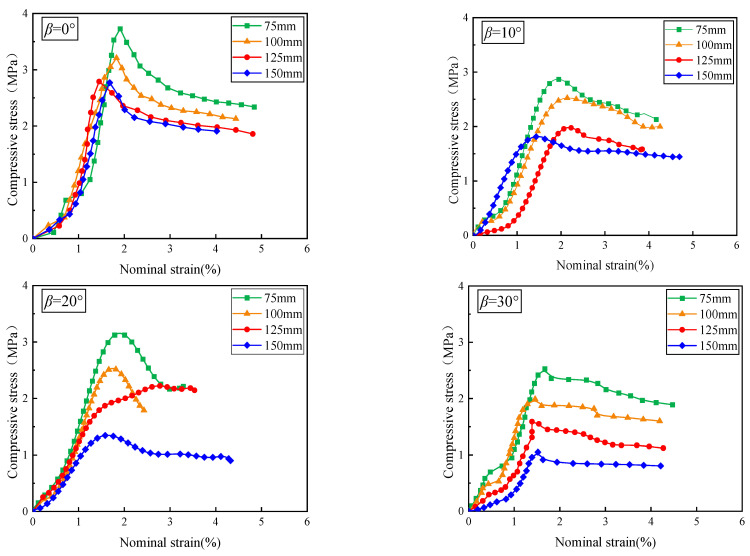

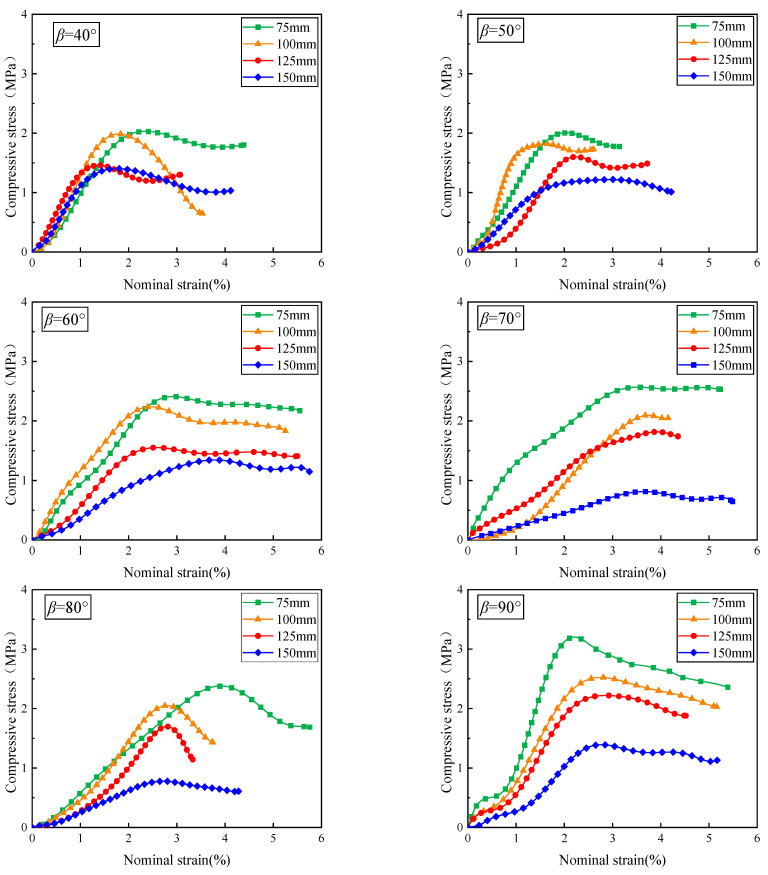
Stress–strain curves of open crack specimens.

Figure 5 shows the relationship between compressive stress and nominal strain for open crack specimens with varying sizes and crack inclination angles. For a given crack inclination angle, the stress–strain curves of specimens with varying sizes exhibit similar trends. Initially, the specimen is in the compaction stage, and the stress–axial strain curve exhibits nonlinear deformation and shows a slow growth trend. The nonlinear deformation is more pronounced in smaller specimens. This nonlinear behavior is more noticeable in smaller specimens. As the test continues, the strain increases progressively, and the specimens enter the elastic deformation phase, where the stress–strain curve shows a clear linear rise, consistent with standard elastic behavior. Once the peak compressive stress is reached, the prefabricated crack expands rapidly, and the stress–strain curve transitions to nonlinear deformation. As the crack continues to propagate, the specimen experiences failure, its compressive strength decreases, and the compressive stress gradually diminishes, ultimately reaching the strain-softening stage. For a constant crack inclination angle *β*, the peak compressive stress decreases as the specimen size increases, demonstrating a clear size effect. Taking *β* = 30° as an example, the peak compressive stresses of 75 mm to 150 mm specimens were 2.530 MPa, 1.990 MPa, 1.590 MPa, and 1.050 MPa, respectively. Compared with that of the 75 mm specimen, the peak compressive stress was reduced by 21.3%, 20.1%, and 33.9%, respectively. These decreases were more pronounced at larger crack inclinations. The peak compressive stress is highest at the crack inclination angle (*β*) of 0° and 90°. This is because when *β* = 0°, the crack is parallel to the direction of compressive stress, and only the transverse stress acts on the crack tip, resulting in tensile stress; meanwhile, when *β* = 90°, the crack surface is perpendicular to the direction of compressive stress, and the crack is only subjected to normal stress, and the sample can withstand greater loads. These observations align with the findings of Wang et al. [18].

**Figure 6:**
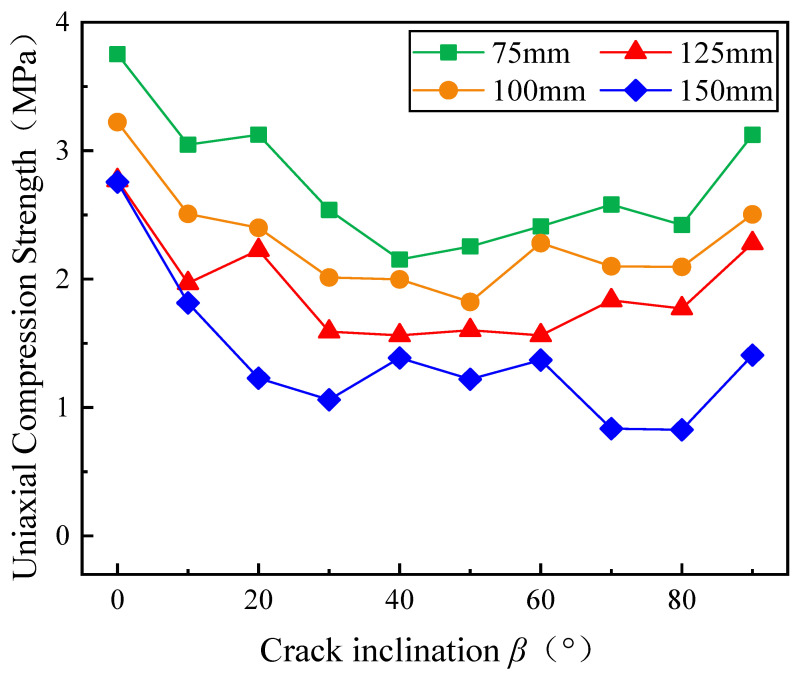
Crack inclusion and uniaxial compressive strength curves of gypsum specimens of different sizes.

Figure 6 illustrates how compressive strength varies with crack inclination angle for specimens of different sizes. It is clear that the compressive strength of specimens with varying sizes follows a similar trend in relation to *β*. When *β* = 0°, the compressive strength is at its highest, with the compressive strength of the 150 mm specimen closely matching that of the 125 mm specimen. As β increases, the compressive strength initially decreases. Within the range *β* of 20° to 80°, the compressive strength begins to fluctuate gradually, and its continuous rising and falling conclude with a sharp rise at *β* = 90°. The overall trend is first decreasing and then increasing, which is similar to the results of many previous studies [18,61,62,63]. For a given crack inclination angle *β*, the compressive strength decreases as specimen size increases. Taking *β* = 50° as an example, the uniaxial compressive strength of 75 mm to 150 mm specimens were 2.254 MPa, 1.821 MPa, 1.601 MPa, and 1.219 MPa, respectively. The differences between specimens with adjacent sizes were 0.433 MPa, 0.219 MPa, and 0.382 MPa, corresponding to reductions of 19.2%, 12.0%, and 23.9%, respectively, as specimen size increased. When *β* = 10°, the compressive strength of 75 mm specimen was 3.046 MPa, while that of 150 mm specimen was 1.231 MPa, a difference of 1.895 MPa, corresponding to a 40.4% decrease in compressive strength. This highlights the significant effect that specimen size has on the compressive strength of gypsum specimens.

### 4.2. Failure Characteristics Analysis

**Figure 7:**
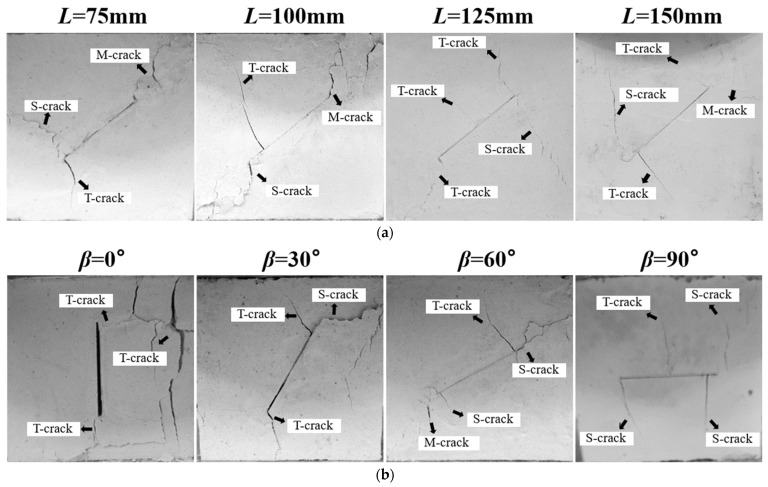
Typical failure characteristics of open crack specimens: (**a**) when *β* = 50°, with specimen sizes ranging from 75 mm to 150 mm; (**b**) when *L* = 75 mm, with *β* ranging from 0° to 90°.

As the test progresses, the prefabricated crack of the specimen will gradually expand into various types of macroscopic cracks as the load increases. Initially, in the early stages of loading, tensile cracks typically form first. As the compressive stress continues to increase, the specimen gradually deforms, and shear cracks, along with mixed-mode cracks, begin to emerge [52,58]. To facilitate crack type analysis, Figure 7 includes annotations: tensile cracks are labeled as T-cracks, shear cracks as S-cracks, and mixed tensile-shear cracks as M-cracks. At the same prefabricated crack inclination (*β*), the failure characteristics of specimens across different sizes are generally consistent. Thus, the failure process at *β* = 50° is presented as a representative example, as shown in Figure 7a. The crack propagation paths are notably similar. In the initial loading stage, the small normal stress is insufficient to compact the crack, leaving it open. Due to the normal stress concentration at the crack tip, smooth tensile cracks first appear. As loading progresses, the normal stress increases, the original crack gradually closes, and shear stress becomes active, causing relative sliding along the fracture surface and leading to near-parallel or perpendicular shear cracks at the original crack tip. For specimen sizes *L* of 75 mm and 100 mm, debris shed from the shear cracks and the expanded cracks became jagged, penetrating the specimen at failure. For specimen sizes *L* of 125 mm and 150 mm, no debris shedding occurred, and the crack propagation paths became smoother with increasing size. This indicates that smaller specimens experience more severe damage.

The failure process at *L* = 75 mm is presented as a representative example, as shown in Figure 7b. *β* clearly affected the crack propagation path. When *β* = 0°, the prefabricated crack remained open at failure as the normal and tangential stresses were zero, leaving only the tangential stress acts on the crack tip, causing tensile cracks to form at the crack tip. The failure occurred directly along the crack, and the sample edge suddenly cracked. As *β* increased, the normal and tangential stresses gradually increased and acted on the crack surface. Normal stress promotes stress concentration at the crack tip, resulting in tensile cracks, while tangential stress induces relative sliding along the crack surface, generating shear cracks at the crack tip. When *β* = 30° and 60°, smooth tensile cracks appeared at the prefabricated crack tip, and the crack inclination angle (*θ*) gradually increased. Simultaneously, shear cracks formed, accompanied by debris shedding. Shear cracks are rougher than tensile cracks, exhibiting jagged paths that traverse the specimen. As *β* further increased, the peak circumferential stress at the crack tip approached zero, preventing tensile crack initiation at the crack tip. When *β* = 90°, only normal stress acted on the crack surface during loading, with shear crack formation primarily due to sliding failure at the crack tip. Microcracks in the middle section were due to the tensile stress between crack surfaces under distributed load during the early loading stage when the crack remained open, causing tensile microcracks to develop in the middle part of the crack [61,64].

**Figure 8:**
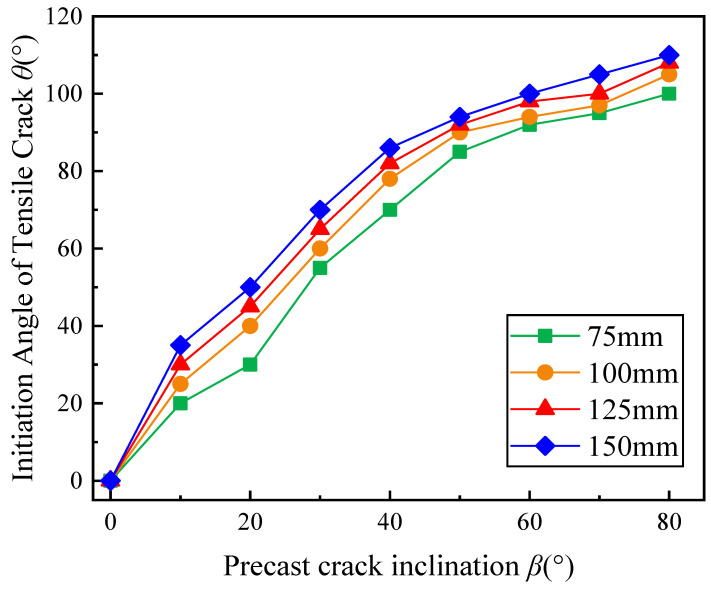
Influence of *β* on *θ* in gypsum specimens of different sizes.

Figure 8 presents the relationship between *θ* and *β* for gypsum specimens with open cracks of varying sizes, showing that as *β* increases, *θ* of all sizes gradually increases. With *β* = 60°, the curve for all specimen sizes gradually stabilized. In addition, the *θ* value of a larger specimen was greater under the same *β* in the range of 10° to 80°. Taking *β* = 30° as an example, the *θ* for specimens of 75 mm to 150 mm were 55°, 60°, 65° and 70°, respectively, indicating that *θ* was influenced by specimen size to some extent. It should be noticed that with *β* = 90°, where $\sigma_{\theta }=0$, no tensile cracks initiation occurred at the crack tip. Thus, this point was eliminated.

### 4.3. Theoretical Analysis of Crack Initiation Angle Under Compression-Shear Stress

First, the relative openness *η* of specimens with side lengths of 75 mm to 150 mm were calculated by equation $\eta =\sqrt{b/a}$ as 0.163, 0.141, 0.126, and 0.115, respectively. The relationship between the *β* and *θ* for five relative critical sizes (*α*) was then plotted based on Equation (11). Meanwhile, the MTS criterion prediction curve was added to the diagram for comparison.

**Figure 9:**
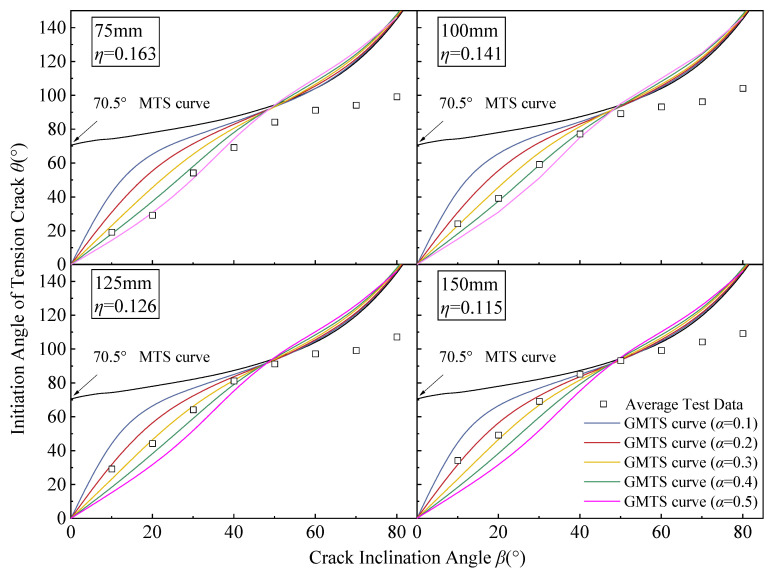
GMTS tension crack initiation angle prediction curves and average test data for gypsum specimens with different *η* values.

According to Figure 9, *θ* of specimens with different sizes gradually increased as *β* increased, while *θ* predicted by the MTS criterion not considering T-stress was always above 70.5°. With *β* below 50°, the MTS criterion theoretical values differed significantly from the test values. With the GMTS criterion considering T-stress, the test values became consistent with the GMTS criterion theoretical curves of different *α*, with a significantly improved degree of consistency in the predicted results. The test values for specimens with a size *L* of 75 to 150 mm are in the highest agreement with the theoretical curve with the critical size *α* = 0.5, *α* = 0.4, *α* = 0.3, and *α* = 0.2. Therefore, with increased specimen size, the *α* value of the GMTS theoretical curve matching the test values gradually decreased, indicating that this criterion can reflect the size differences in the open crack specimens. In the meantime, the theoretical *θ* increased with the decrease in *α* at the same specimen size. With *β* = 50°, the MTS theory and the GMTS theory predict basically the same *θ* for specimens of all sizes, all around 90°. As *β* exceeds 50°, the MTS criterion theoretical curves and the GMTS criterion theoretical curves gradually become the same. As *β* increases, theoretical *θ* values rise consistently but show a growing deviation from experimental measurements. At the same specimen size, the theoretical *θ* increases as *α* decreases, unlike the cases with *β* below 50°. It is important to highlight that the theoretical *θ* is calculated to be 180° when *β* is 90°, which is a digital limit value that will not appear in actual situations. Hence, it was eliminated from the comparative analysis.

With *β* between 0° and 50°, the theoretical *θ* closely matched the test value. However, as *β* increased beyond this range, the theoretical *θ* progressively exceeded the observed test values. With the increase in *β*, the deviation degree gradually increased. However, specimens of different sizes showed varying deviation degrees, with lower deviation degrees in larger specimens. With *β* = 60°, the *θ* test values of the 150 mm to 75 mm specimens were 100°, 98°, 94°, and 92°, respectively, constituting a 3°, 5°, 9°, and 11° difference with the MTS criterion theoretical *θ* value of 103°, respectively. The deviation increased with specimen size, suggesting that larger specimens tend to have smaller discrepancies between the theoretical and observed values. This deviation between theoretical and experimental results can be attributed to the assumptions inherent in fracture mechanics theories, which generally assume crack initiation at the crack tip. Both the GMTS criterion and MTS criterion reflect the stress concentration characteristics at the crack end. As *β* increased, however, the crack initiation position deviated increasingly from the crack tip. This resulted in a growing disparity between the theoretical predictions and experimental observations. Furthermore, with the same *β*, the crack tip of larger specimens tended to expand earlier due to greater tangential stress on it, with lower deviation degrees.

### 4.4. Circumferential Stress at the Crack Under Compression-Shear Stress

**Figure 10:**
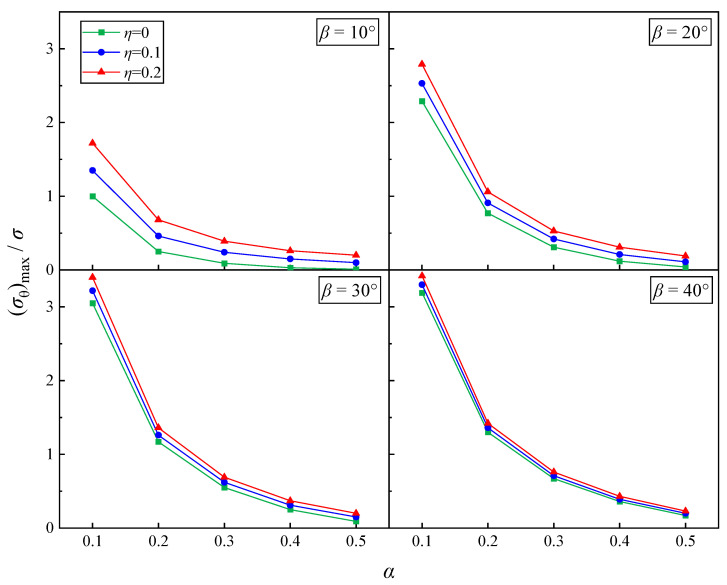
The relationship between *α* and ${{(\sigma }_{\theta })}_{\max}$/*σ* with different *β*.

Figure 10 shows the variation in the maximum circumferential stress ${{(\sigma }_{\theta })}_{\max}$ with *α* for three different passivation coefficients *η*. The maximum circumferential stress was analyzed in a dimensionless manner. The analysis covers inclination angles *β* = 10°, 20°, 30°, and 40°, with *η* = 0, 0.1, and 0.2. From the figure, it is apparent that both for mathematical cracks and open cracks, ${{(\sigma }_{\theta })}_{\max}$ decreases as *α* increases for different *β*. This indicates that, for the same *β*, larger specimens experience greater circumferential stress under compressive-shear stress, resulting in lower stress requirements for crack failure. Additionally, at a fixed *α*, specimens with a higher *η* exhibit larger ${{(\sigma }_{\theta })}_{\max}$, indicating that the degree of crack opening also influences the cracking stress. As *β* increases, ${{(\sigma }_{\theta })}_{\max}$ gradually increases, and the values for different *η* approach each other. This behavior can be attributed to the growing normal stress acting on the crack as *β* increases. In practical experiments, as loading continues, the cracks gradually close, and shear stress becomes the dominant factor controlling crack tip failure.

### 4.5. Fracture Process Zone Analysis of Open Crack Specimens Under Compression-Shear Stress

**Figure 11:**
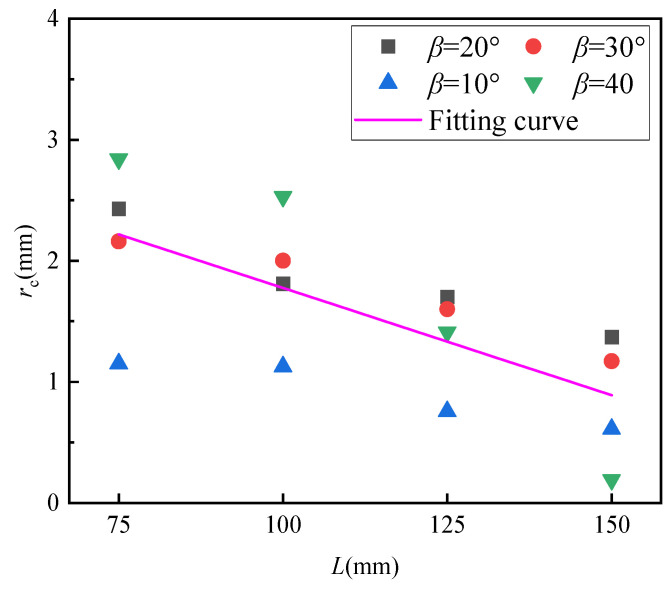
The relationship between *r*_c_ and gypsum specimen size under three typical *β* values.

The following steps were performed to better analyze the fracture process zone (FPZ), also known as the critical crack tip size *(r*_c_), First, four typical angles (*β* = 10°, *β* = 20°, *β* = 30°, and *β* = 40°) were chosen. These angles were selected from a range where the theoretical predictions and experimental measurements align closely, specifically *β* < 50°. The matching relative critical size *α* was estimated based on the test values in Figure 9. The value of *r*_c_ was then calculated using Equation $\alpha =\sqrt{2r_{c}/a}$, as shown in Table 2. The results indicate that *r*_c_ is notably influenced by the specimen size. This finding contrasts with previous studies, which treated *r*_c_ as an inherent material property [65]. As shown in Figure 11, a plot was created to illustrate the relationship between *r*_c_ and specimen size for different *β*, and a linear relationship between *r*_c_ and *L* was fitted: $r_{c}=-0.01772L+3.54648$ (*R*^2^ = 0.45622). It was observed that *r*_c_ decreases as specimen size increases, a trend that aligns with the results reported by Wang et al. [18]. This size-dependent behavior of *r*_c_ can be understood from an energy perspective. The FPZ serves as an energy-absorbing region, and this area in quasi-brittle materials is filled with microcracks. The applied stress at the crack tip is primarily converted into two forms of energy: the elastic strain energy of the specimen and the energy necessary to develop the fracture process zone (FPZ). Therefore, a certain load is required to fully form the FPZ. An increase in the length of the FPZ suggests a greater capacity for the crack tip region to absorb and store energy, leading to a size effect. Larger quasi-brittle material specimens have a smaller FPZ that can absorb less energy, making them more prone to failure.

**Figure:**
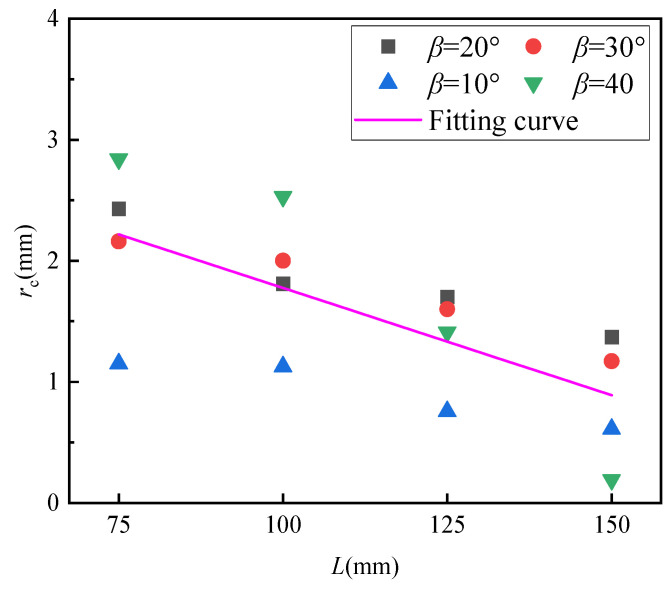


## 5. Conclusions

The results of this study on the fracture behavior and size effect of crack-containing brittle materials under compression-shear conditions have significant practical applications, particularly in industries such as construction, mining, and geotechnical engineering, where predicting material failure is essential. This research provides valuable guidance for the design of engineering structures and the selection of materials. For instance, in geotechnical engineering, understanding crack propagation characteristics aids in predicting rock mass cracking behavior and instability risks, thereby optimizing slope stability design and underground excavation support strategies.

This study provides an examination of how the fracture characteristics of quasi-brittle materials are influenced by the size of the specimen under compression-shear loading conditions. By making gypsum samples with open cracks of varying sizes for uniaxial compression tests, the research examined the propagation behavior of surface cracks in these samples. The primary objective was to establish a clear link between specimen dimensions and fracture behavior, highlighting the size effect in quasi-brittle materials, and the following conclusions were reached:

(1) Open crack gypsum specimens of different sizes produce similar stress–strain curves that initially rises slowly in a nonlinear fashion, followed by a linear increase. After reaching the peak stress, the specimen fails, and the curve drops and gradually stabilizes. The uniaxial compressive strength of the specimens exhibits a significant size effect, with smaller specimens exhibiting higher compressive strength.
(2) The open crack specimens usually produce tension cracks first, and specimens of different sizes exhibit similar crack propagation paths which are strongly influenced by *β*. As *β* increases *θ* gradually increases. At *β* = 90°, shear failure is observed to occur. When specimens of the same *β* are compared, an increase in specimen size leads to a corresponding rise in *θ*, indicating size effect.
(3) With *β* below 50°, the MTS theoretical curve differs significantly from the test value, while the GMTS theoretical curves of smaller *α* are consistent with the test values of lager specimens. As *β* increases, the theoretical curve gradually exceeds the test value, and the deviation degree gradually increases.
(4) For the same *β*, larger specimens had higher circumferential stress, requiring less stress to reach failure. The decrease in the effect of the passivation coefficient (*η*) on circumferential stress as β increases indicates that the role of *η* becomes less significant at higher *β* values.
(5) Under uniaxial compression, *r_c_* decreases with increasing specimen size, fitting a linear relationship: $r_{c}=-0.01772L+3.54648$ (*R*^2^ = 0.45622). From an energy perspective, the FPZ serves as a crucial energy-absorbing zone that influences fracture behavior, indicating that the size effect is a vital consideration in the design and assessment of quasi-brittle materials under compression-shear stress.

## Figures and Tables

**Table 1:** Specimen testing scheme.

*L*/(mm)	2*b*/(mm)	2*a*/(mm)	*β/*(°)
75	1	37.5	0, 10, 20, 30, 40, 50, 60, 70, 80, 90
100		50	
125		62.5	
150		75	

**Table 2:** The *r*_c_ value of a typical angle.

*L*/mm	*β*/°			
	10	20	30	40
75	1.15	2.43	2.16	2.84
100	1.13	1.81	2.00	2.53
125	0.76	1.70	1.60	1.41
150	0.61	1.37	1.17	0.19

## Data Availability

The data that support the findings of this study are available within the manuscript.
